# Manganese Phosphate Self-assembled Nanoparticle Surface and Its application for Superoxide Anion Detection

**DOI:** 10.1038/srep28989

**Published:** 2016-06-30

**Authors:** Xiaohui Shen, Qi Wang, Yuhong Liu, Wenxiao Xue, Lie Ma, Shuaihui Feng, Mimi Wan, Fenghe Wang, Chun Mao

**Affiliations:** 1 National and Local Joint Engineering Research Center of Biomedical Functional Materials, Jiangsu Key Laboratory of Biofunctional Materials, School of Chemistry and Materials Science, Nanjing Normal University, Nanjing, 210023, China

## Abstract

Quantitative analysis of superoxide anion (O_2_^·−^) has increasing importance considering its potential damages to organism. Herein, a novel Mn-superoxide dismutase (MnSOD) mimics, silica-manganous phosphate (SiO_2_-Mn_3_(PO_4_)_2_) nanoparticles, were designed and synthesized by surface self-assembly processes that occur on the surface of silica-phytic acid (SiO_2_-PA) nanoparticles. The composite nanoparticles were characterized by fourier transform infrared spectroscopy (FTIR), transmission electron microscopy (TEM), scanning electronic microscopy (SEM), electron diffraction pattern, energy dispersive spectroscopy (EDS) and elemental mapping. Then the electrochemical measurements of O_2_^·−^ based on the incorporation of SiO_2_-Mn_3_(PO_4_)_2_ onto the surface of electrodes were performed, and some satisfactory results were obtained. This is the first report that manganous phosphate (Mn_3_(PO_4_)_2_) nanoparticles with shape-controlled, but not multilayer sheets, were utilized for O_2_^·−^ detection. The surface self-assembly technology we proposed will offer the ideal material to construct more types biosensor and catalytic system for its nanosized effect.

Active reactive oxygen species (ROS) containing oxygen atoms are the substances with strong oxidizing ability, which can cause or aggravate cancer, cardiovascular diseases, asthma, cataract, ulcer disease, Alzheimer’s disease, Parkinson’s disease and other diseases. O_2_^·−^, the critical important part of the so-called ROS, is implicated in many physiological and pathological processes^1^,^2^,^3^. Under normal physiological conditions, O_2_^·−^ maintains the relatively balanced level *in vivo*. Once the cell produces excessive O_2_^·−^ in response to external stimulus or pathological changes, it will lead to etiology of aging, cancer, and progressive neurodegenerative diseases such as Parkinson’s disease. Thus, real-time analysis and detection of O_2_^·−^ have great significance. A variety of approaches have been tried to measure O_2_^·−^ concentration, such as electron spin resonance^4^,^5^,^6^, spectrophotometry^7^, chemiluminescence^8^, colorimetry^9^,^10^, chromatograph^11^,^12^ and fluorescence^13^,^14^,^15^. However, these methods cost much and usually occupy too much space. In comparison with other methods, the electrochemical method has recently attracted a great deal of attention owing to its advantages including high sensitivity, low detection limit, simplicity, direct, real-time detection and so on.

Up to date, the commonly used electrochemical enzyme sensors are fabricated by immobilizing superoxide dismutase (SOD) and cytochrome (cyt c) onto the electrode surface. However, the enzymatic O_2_^·−^ sensors are easily affected by pH and temperature changes, which limit their practical applications due to the poor stability of nature enzyme. Nanozymes, possessing enzymatic activities with nanostructure, have attracted particular attention as emerging natural enzyme mimics, they offer the possibility of lowered cost, improved stability, and excellent recyclability^16^,^17^,^18^. Meanwhile, bionic concept has gained more and more attention^19^,^20^,^21^. Mn-superoxide dismutase (MnSOD) mimics, manganous phosphate (Mn_3_(PO_4_)_2_), manganous pyrophosphate (Mn_2_P_2_O_7_) and manganese (II) complexes are usually used to fabricate biosensors for O_2_^·−^ detection^22^,^23^. Cabelli have studied the antioxidant mechanism of aggregated Mn_3_(PO_4_)_2_ particles in organic vivo^24^. Li used DNA as a template to produce Mn_3_(PO_4_)_2_ nanosheets and decorated this biomimetic enzyme onto the electrode surface for sensitive *in-situ* detection of O_2_^·− ^25. However, the intrinsic drawbacks of DNA, including high cost, instability, and storage difficulty, may limit their widely applications of electrochemical sensors. Dai also reported the high efficient catalysis of Mn_2_P_2_O_7_, which was used as a SOD mimic for O_2_^·−^ detection^26^. There is a serious problem in dealing with the preparation of these reported MnSOD mimics. It is that the conventional synthesized MnSOD mimics that reported in the previous literatures have multilayer sheet structure with uncontrolled shape, thickness and size. This approach will bring resources waste and low catalytic efficiency. We wonder how it is possible to utilize surface self-assembly technology and nanotechnology to construct a more efficient MnSOD mimic for promoting analytical properties.

In this paper, SiO_2_-Mn_3_(PO_4_)_2_ NPs were synthesized by surface self-assembly processes that occur on the surface of SiO_2_-phytic acid (SiO_2_-PA). To the best of our knowledge, there are no reports employing surface coating technique to immobilize Mn_3_(PO_4_)_2_ onto the surface of NPs for O_2_^·−^ detection. The SiO_2_-Mn_3_(PO_4_)_2_ NPs have many advantages, like controllable shape with nanoscale, high specificsurface area than that of nano-sheet structure, low cost, simple preparation process, non-toxic, and so on. This novel MnSOD mimic we prepared is utilized to fabricate biosensors, and the electrochemical measurements of O_2_^·−^ based on the incorporation of SiO_2_-Mn_3_(PO_4_)_2_ onto the electrodes surface are performed.

## Results and Discussion

**Figure 1:**
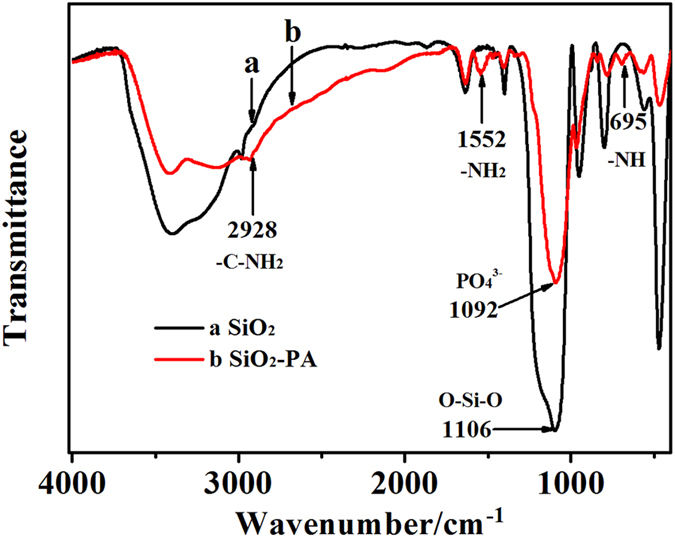
FTIR spectra of (**a**) SiO_2_ NPs, and (**b**) SiO_2_-PA NPs.

Figure 1 showed the fourier transform infrared (FTIR) spectroscopy of SiO_2_ NPs (a) and SiO_2_-PA NPs (b). For curve (a), the appearance of characteristic peak at 1106 cm^−1^ and 957 cm^−1^ were attributed to the O-Si-O bonds stretching vibration, indicating that SiO_2_ NPs were successfully synthesized^27^. Compared with unmodified SiO_2_ NPs, the SiO_2_-PA NPs illustrated three extra peaks at 2928, 1552 and 695 cm^−1^, which should be attributed to -C-NH_2_ stretching, symmetric -NH_2_ stretching, and the bending vibrations of -NH in APTES, respectively^28^. The results indicated that APTES was successfully modified onto the surface of SiO_2_ NPs^29^. More importantly, an adsorption peak at 1092 cm^−1^ was observed due to the overlap of the characteristic peak of phosphate group (PO_4_^3−^) and the peak of asymmetric O-Si-O stretching^30^. The results confirmed that the SiO_2_ NPs were successfully modified by APTES and PA.

**Figure 2:**
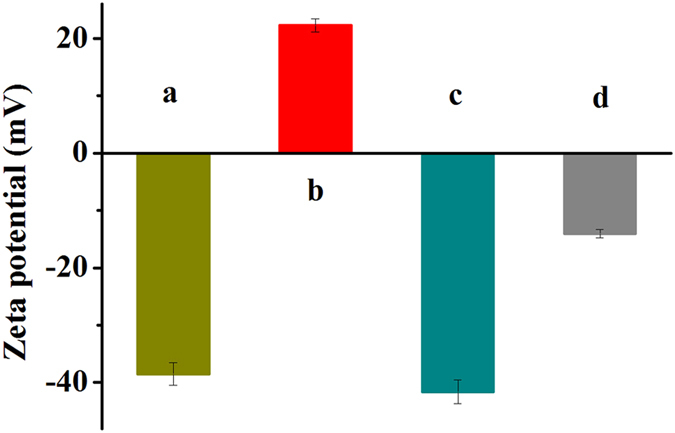
(**a**) SiO_2_ NPs, (**b**) SiO_2_-NH_2_ NPs, (**c**) SiO_2_-PA NPs, and (**d**) SiO_2_-Mn_3_(PO_4_)_2_ NPs of Zeta potential analysis.

As shown in Fig. 2a, the Zeta potential of SiO_2_ surface was −38.5 mV, which was attributed to many -OH and other oxygen-containing groups that present in the SiO_2_ NPs surface. When modified with APTES, the Zeta potential of APTES-SiO_2_ NPs increased to +22.3 mV that due to the amine groups on the surface of the particles (Fig. 2b). However, the Zeta potential measurements for SiO_2_-PA NPs (Fig. 2c) showed a negative surface charge that owing to the six phosphate groups of PA. When Mn^2+^ ions in solution were self-assembled onto the surface of SiO_2_-PA NPs, the zeta potential increased to −14.1 mV. The change of Zeta potential indicated that SiO_2_-Mn_3_(PO_4_)_2_ NPs were successfully synthesized by self-assembly technology based on the electrostatic interaction that between Mn^2+^ ions and the phosphate groups^31^.

**Figure 3:**
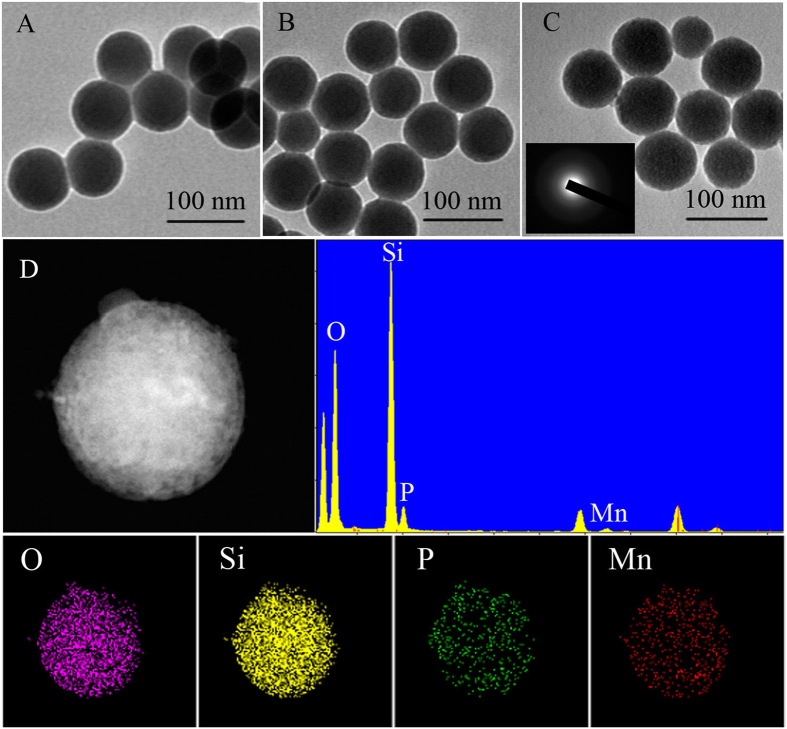
TEM images of (**A**) SiO_2_ NPs, (**B**) SiO_2_-PA NPs, (**C**) SiO_2_-Mn_3_(PO_4_)_2_ NPs, and (**D**) EDS spectrum and elemental mapping of SiO_2_-Mn_3_(PO_4_)_2_ NPs.

**Figure 4:**
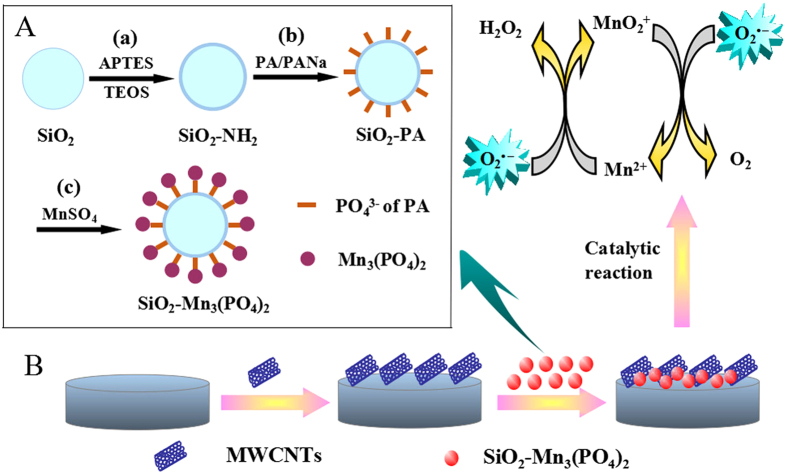
(**A**) Schematic illustration of the formation of SiO_2_-Mn_3_(PO_4_)_2_ NPs: (a) Amino-modified process of SiO_2_ NPs, (b) Phytic acid modified process of SiO_2_-NH_2_ NPs, and (c) Self-assembled of Mn_3_(PO_4_)_2_ on the outer surface of SiO_2_-PA NPs, (**B**) Schematic diagram for the construction of the biosensor.

The TEM and SEM images were also employed to further confirm the formation of SiO_2_-Mn_3_(PO_4_)_2_ NPs. Figure 3A revealed that the spherical SiO_2_ NPs were obtained with the average particle size of 75 nm. After surface self-assembly of PA and Mn^2+^ sequentially, the two sizes of SiO_2_-PA NPs and SiO_2_-Mn_3_(PO_4_)_2_ NPs showed a slight increase (Fig. 3B,C), respectively. Furthermore, the electron diffraction pattern displayed an amorphous diffraction pattern of Mn_3_(PO_4_)_2_ that deposited on the surface of silica (see the inset from Fig. 3C). And the corresponding elemental mapping of oxygen (O), silicon (Si), phosphorus (P), and manganese (Mn) from the SiO_2_-Mn_3_(PO_4_)_2_ NPs were indicated in Fig. 3D. The energy dispersive spectroscopy (EDS) of SiO_2_-Mn_3_(PO_4_)_2_ NPs showed that the different atomic percentages were 85.32% (O), 13.10% (Si), 1.47% (P), and 0.11% (Mn), respectively. It can be concluded that Mn_3_(PO_4_)_2_ was firmly coated onto the outer surface of the SiO_2_-PA NPs. Mn_3_(PO_4_)_2_ layer has little effect on the size growth of SiO_2_ NPs because it was only monolayer of Mn_3_(PO_4_)_2_ molecular that self-assembled onto the outer surface of SiO_2_ NPs based on the electrostatic interaction. Here, the stability of Mn_3_(PO_4_)_2_ supported on SiO_2_ NPs was evaluated by Zeta potential measurement after three weeks of storage. As was shown in Figure S1, the Zeta potential of the SiO_2_-Mn_3_(PO_4_)_2_ NPs had almost no change after three weeks of storage, indicating the Mn_3_(PO_4_)_2_ NPs have long-term stability. SEM images were also used to investigate the surface texture change after Mn_3_(PO_4_)_2_ coating on SiO_2_ NPs, and the particle size of Mn_3_(PO_4_)_2._ Figure S2 showed the SEM images of the SiO_2_ NPs, SiO_2_-PA NPs and SiO_2_-Mn_3_(PO_4_)_2_ NPs, respectively. It can be observed from the SEM results that the samples with Mn_3_(PO_4_)_2_ coating can remain its original spherical morphology. Meantime, it can also be seen in this figure that particle size of the samples showed a slight increase after Mn_3_(PO_4_)_2_ coating on SiO_2_ NPs, which was in consistence with the results obtained by TEM images as showed in Fig. 3. Figure 4A illustrated the synthesis process of SiO_2_-Mn_3_(PO_4_)_2_ NPs. The formational mechanism of this biomimetic enzyme could be explained as follows: After dropping into MnSO_4_ solution, PO_4_^3−^ ions, derived from the surface of SiO_2_-PA NPs, were in combination with Mn^2+^ions by electrostatic interaction to form Mn_3_(PO_4_)_2_. When the PO_4_^3−^ ions were consumed, the monolayer of Mn_3_(PO_4_)_2_ molecular was self-assembly on the outer surface of SiO_2_-PA NPs with controllable morphology. In addition, only aggregated Mn_3_(PO_4_)_2_ particles were observed in the absence of SiO_2_ NPs with the same reaction conditions (Supplementary Figure S3).

**Figure 5:**
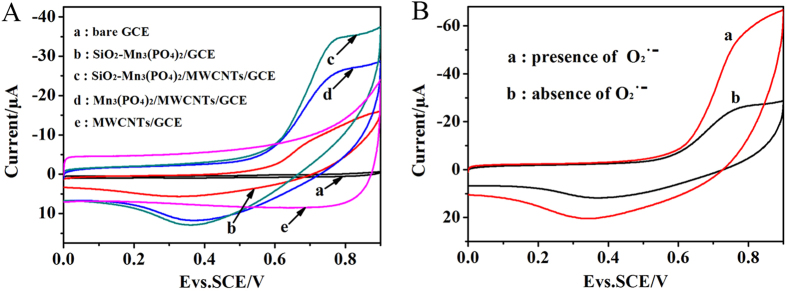
(**A**) Cyclic voltammograms (CVs) of (a) bare GCE, (b) SiO_2_-Mn_3_(PO_4_)_2_/GCE, (**c**) SiO_2_-Mn_3_(PO_4_)_2_/MWCNTs/GCE, (d) Mn_3_(PO_4_)_2_/MWCNTs/GCE, and (e) MWCNTs/GCE, (**B**) CVs of SiO_2_-Mn_3_(PO_4_)_2_/MWCNTs/GCE in the presence (a) and absence (b) of 1.0 μmol L^−1^ O_2_^·−^ in PBS (pH 7.4), scan rate: 100 mV·s^−1^.

A schematic drawing of the stepwise construction process of modified glassy carbon electrode (GCE) was described in Fig. 4B. The electrochemical properties of the SiO_2_-Mn_3_(PO_4_)_2_/Multi-walled carbon nanotubes (MWCNTs)/GCE were investigated by cyclic voltammetry (CV) and Electrochemical Impedance Spectroscopy (EIS)^32^,^33^,^34^,^35^ (Supplementary Figure S4). Figure 5A displayed that all fabrication process of SiO_2_-Mn_3_(PO_4_)_2_/MWCNTs/GCE were carried out by CV in nitrogen saturated phosphate buffered solution (PBS) at a scan rate of 100 mV·s^−1^. In the working potential range of 0–0.9 V, there was no electrochemical signal can be observed at the bare GCE (curve a). In contrast, the SiO_2_-Mn_3_(PO_4_)_2_/GCE exhibited a pair of weakly redox peaks (curve b). When the MWCNTs/GCE was modified with SiO_2_-Mn_3_(PO_4_)_2_ NPs, the oxidation-reduction peaks were more obviously observed (curve c) that due to the excellent electronic conductivity of MWCNTs (Supplementary Figure S5), and the sensitivity of this biosensor was largely improved^36^. Moreover, the peak currents of SiO_2_-Mn_3_(PO_4_)_2_/MWCNTs/GCE (curve c) were much larger than that of Mn_3_(PO_4_)_2_/MWCNTs/GCE (curve d). Results demonstrated that the electro-catalytic effect of SiO_2_-Mn_3_(PO_4_)_2_ was much higher than that of Mn_3_(PO_4_)_2_ aggregated particles. It can be attributed to that the nanosized SiO_2_-Mn_3_(PO_4_)_2_ possessed high specific surface area. As a result, it will help improve the catalytic efficiency of O_2_^·−^ in the electrolyte. When the bare GCE was only modified with MWCNTs, the background current was more clearly observed (curve e).

To study the catalysis effect of the SiO_2_-Mn_3_(PO_4_)_2_ NPs, the biosensor in PBS and PBS of containing 1.0 μmol L^−1^ O_2_^·−^ were measured by CV, respectively. As shown in Fig. 5B, in the PBS containing of 1.0 μmol L^−1^ O_2_^·−^ (curve a), both anodic and cathodic peak currents that corresponding to the redox reaction of in PBS (curve b) clearly increased that can be attributed to the oxidation and reduction of O_2_^·−^, respectively^37^. According to the previous reports^38^,^39^, O_2_^·−^ was converted into O_2_ and H_2_O_2_ during the disproportionation reaction that O_2_^·−^ was catalyzed by Mn^2+^ in PBS. In the anodic process, O_2_^·−^ was oxidized to O_2_ by the oxidation effect of MnO_2_^+^, while MnO_2_^+^ was reduced to Mn^2+^. On the contrary, Mn^2+^ was oxidized to MnO_2_^+^ in the cathodic process. As demonstrated above, the SiO_2_-Mn_3_(PO_4_)_2_/MWCNTs/GCE can be applied to detect O_2_^·−^ by measuring the oxidation or reduction currents because of the high efficient catalysis of SiO_2_-Mn_3_(PO_4_)_2_. To further prove this proposed mechanism/reaction, X-ray photoelectron spectroscopy (XPS) analysis was carried out to analyze the composition and chemical configuration of the SiO_2_-Mn_3_(PO_4_)_2_ NPs before and after electrocatalysis process. More details about the XPS spectra of Mn 2p were presented in Figure S6.

**Figure 6:**
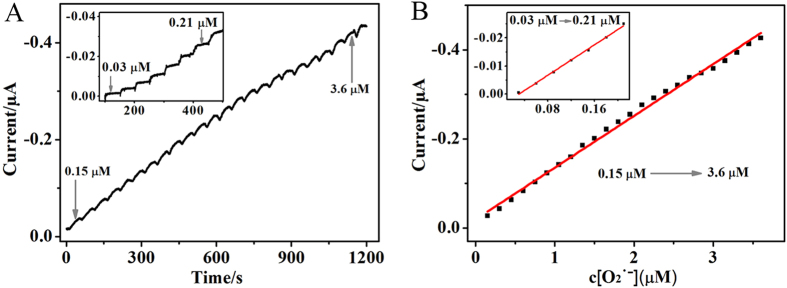
(**A**) Typical amperometric curve recorded at 0.484 V in PBS (pH 7.4) for SiO_2_-Mn_3_(PO_4_)_2_/MWCNTs/GCE, From 0.03 to 0.21 μM, the O_2_^·−^ concentration of each adding step was 0.03 μM, from 0.15 to 3.6 μM, each adding step was 0.15 μM, (**B**) Linear calibration plot of the response current vs the O_2_^·−^ concentration.

The typical current-time plot of SiO_2_-Mn_3_(PO_4_)_2_/MWCNTs/GCE at the applied potential of 0.484 V upon successive additions of O_2_^·−^ was provided in Fig. 6A. In this experiment, while being stirred, O_2_^·−^ solution was added once per 50 seconds. With the injection of O_2_^·−^, the response of this biosensor rapidly achieved 95% of the steady-state current within 2.9 s (Supplementary Figure S7). The SiO_2_-Mn_3_(PO_4_)_2_/MWCNTs/GCE showed a wide linear range from 0.03 to 0.21 μM and 0.15 to 3.6 μM with the correlation coefficient of 0.9966 and 0.9959, respectively. The relation of the oxidation peak current *vs* the concentration of O_2_^·−^ was linear with a detection limit of 0.0175 μM (S/N = 3). The biosensor exhibited more excellent performance than some O_2_^·−^ biosensors that reported by the previous papers using different electrode materials, such as SOD, Mn_3_(PO_4_)_2_, and Mn_2_P_2_O_7_ (Supplementary Table 1). The corresponding calibration curves for O_2_^·−^ were depicted in Fig. 6B. The linear equations were i (μA) = 0.00418 − 0.1358c (μM) and i (μA) = −0.01932 − 0.1263c (μM), respectively. The biosensor was applied to the detection of O_2_^·−^ and displayed excellent electrochemical behavior.

To verify the applicability of SiO_2_-Mn_3_(PO_4_)_2_ NPs in detection of O_2_^·−^, Xanthine/Xanthine Oxidase (XAN/XOD) was selected to generate O_2_^·−^ (Supplementary Figure S8). In addition, the response of SiO_2_-Mn_3_(PO_4_)_2_/MWCNTs/GCE toward O_2_^·−^ generated by XAN/XOD was investigated by amperometric measurements^40^. As shown in Figure S9, with successive additions of XAN to the solution, a stepwise increase of the current response was observed.

**Figure 7:**
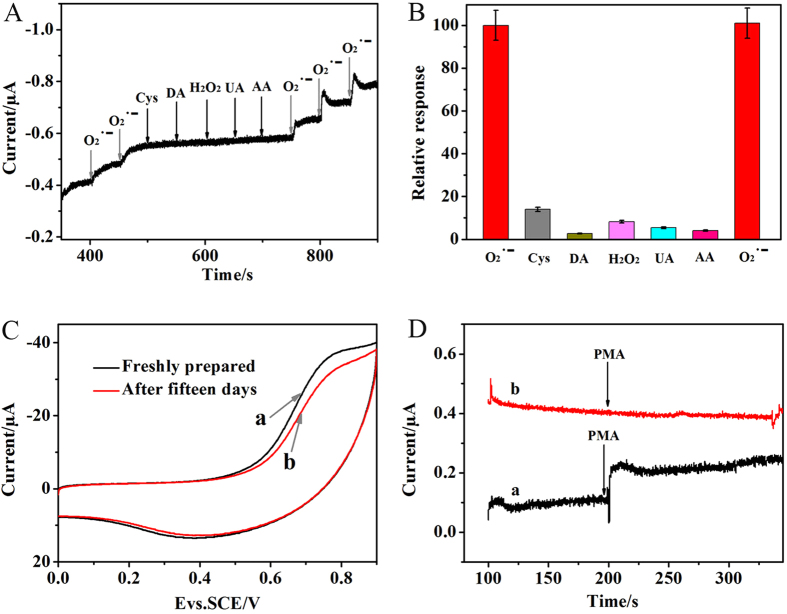
(**A**) Interference test of SiO_2_-Mn_3_(PO_4_)_2_/MWCNTs/GCE, (**B**) The relative response in PBS (pH = 7.4) at 1.0 μM O_2_^·−^, 5.0 μM Cys, 5.0 μM DA, 10 μM H_2_O_2_, 10 μM UA and 10 μM AA, (**C**) CVs of SiO_2_-Mn_3_(PO_4_)_2_/MWCNTs/GCE, (a) Freshly prepared, and (b) after fifteen days in 0.1 M PBS, Scan rate was 100 mV·s^−1^, and (**D**) Amperometric response of the SiO_2_-Mn_3_(PO_4_)_2_/MWCNTs/GCE in the (a) presence, and (b) absence of HeLa cells upon sequential additions of PMA to 0.1 M PBS at 0.484 V.

To evaluate the anti-interference performance of detecting O_2_^·−^, the biosensor was examined by successive additions of O_2_^·−^ and interfering substances into a 0.1 M PBS at 0.484 V. Figure 7A indicated that there were obviously current responses of the biosensor during the addition of 1.0 μM O_2_^·−^, while no obviously current response could be observed with the addition of the interferences. With the sequential addition of 5.0 μM Cys, 5.0 μM DA, 10 μM H_2_O_2_, 10 μM UA and 10 μM AA, the detection current showed changes of 14%, 2.7%, 8.2%, 5.4% and 4.1% with comparison of 1.0 μM O_2_^·−^, respectively (Fig. 7B). Figure S10 displayed the amperometric response of the SiO_2_-Mn_3_(PO_4_)_2_/MWCNTs/GCE by successive additions of 2.0 μM 18-crown-6 once per 50 seconds. Results demonstrated that this biosensor can eliminate the interference and show an excellent selectivity for detection of O_2_^·−^. To verify the stability, the biosensor was monitored after being stored for three weeks in a refrigerator. Figure 7C indicated that the current response was no apparent decrease, which was much longer than those obtained for enzyme-based O_2_^·−^ biosensors^41^,^42^,^43^,^44^. In order to investigate the binding firmness of SiO_2_-Mn_3_(PO_4_)_2_ NPs decorated onto the MWCNTs/GCE electrode, the reuse ability of the SiO_2_-Mn_3_(PO_4_)_2_/MWCNTs/GCE electrode was tested. Figure S11 displayed the CV curves of the biosensor for 20 cycles, which showed almost overlap curves, indicating the biosensor we prepared had a good cycle stability that can attributed to the good binding state of the SiO_2_-Mn_3_(PO_4_)_2_ NPs and the MWCNTs/GCE electrode.

Real-time detection performance of the biomimetic enzyme sensor has also been monitored by detecting O_2_^·−^ released from the HeLa cells. The amperometric responses of the biosensor were obtained at applied potentials of 0.484 V versus Ag/AgCl in 2 mL 0.1 M PBS (pH 7.4) containing 0.5 × 10^5^ cells·mL^−1^. After the injection of 4 μg mL^−1^ phorbol 12-myristate 13-acetate (PMA), which was reported to generate O_2_^·−^ from live cells^45^, the current gradually increased at SiO_2_-Mn_3_(PO_4_)_2_/MWCNTs modified electrode. In this work, PMA was used as a stimulant for the cell to exude O_2_^·− ^46,47. Figure 7D indicated that the strong current signal (0.01457 μA, curve a) was caused by O_2_^·−^ released from the HeLa cells, considering that SiO_2_-Mn_3_(PO_4_)_2_ could selectively decomposes O_2_^·−^. According to the above linear relationship, the O_2_^·−^ concentration of 0.0765 μM was calculated. Thence, the amount of O_2_^·−^ releasing from per 10^5^ cells was calculated to be 0.153 nmol. Meanwhile, in the absence of the HeLa cells and the presence of the treatment of PMA, no obvious current response can be seen on the screen (curve b). To test the effectiveness of this technology, the biomimetic enzyme sensor has also been used to detect the concentration of O_2_^·−^ in plasma (Supplementary Figure S12).

## Conclusion

In this case, the SiO_2_-Mn_3_(PO_4_)_2_ NPs, synthesized *via* self-assembly technique and nanotechnology, were applied in a biomimetic enzyme biosensor for the detection of O_2_^·−^. Results revealed that SiO_2_-Mn_3_(PO_4_)_2_/MWCNTs/GCE showed high electrocatalytic activity toward O_2_^·−^, lower detection limit and wide detection range. Furthermore, the biosensor that assembled under optimal conditions exhibited high selectivity of O_2_^·−^ in the presence of related interference, such as H_2_O_2_, UA, AA, DA and Cys. Meanwhile, the long-term stability and good reproducibility of this biomimetic enzyme biosensor were proved. Compared with the Mn_3_(PO_4_)_2_ multilayer sheets, the modified GCE of SiO_2_-Mn_3_(PO_4_)_2_ with high specific surface area exhibited more excellent analytical performance. Consequently, the biomimetic enzyme-free sensor was successfully applied to detecting O_2_^·−^ that released from live cells, which holds a great promising platform for the reliable monitoring of major diseases in future.

## Methods

### Materials

Tetraethyl orthosilicate (TEOS) was purchased from Sinopharm Chemical Reagent Co. Ltd. Cetyltrimethylammonium bromide (CTAB) was obtained from Shanghai Lingfeng Chemical Reagent Co. Ltd. (3-Aminopropyl) triethoxysilane (APTES), phytic acid (PA, 70 wt%) solution and phytic acid sodium salt hydrate were received from Aladdin Chemistry Co. Ltd (Shanghai, China). Potassium phosphate tribasic trihydrate (K_3_PO_4_·3H_2_O), manganese sulfate monohydrate (MnSO_4_·H_2_O) and dimethyl sulfoxide (DMSO) were obtained from Sinopharm Chemical Reagent Co. Ltd. Potassium hyperoxide (KO_2_) was purchased from Alfa Aesar. Multi-walled carbon nanotubes (MWCNTs) was purchased from Shenzhen Nanotech Port Co. Ltd. Triton X-100, nafion (5 wt% solution in lower aliphatic alcohol), 18-crown-6, phorbol 12-myristate 13-acetate (PMA), dopamine (DA), cysteine (Cys), ascorbic acid (AA) and uric acid (UA) were acquired from Aladdin Sigma-Aldrich Co. (USA). Hydrogen peroxide (H_2_O_2_, 30%) was received from Beijing Chemical Works (China). Phosphate buffer solution (PBS) was obtained by dissolving 8.0 g NaCl, 0.2 g KCl, 1.44 g NaH_2_PO_4_ and 0.24 g KH_2_PO_4_ in 1000 mL double-distilled water.

### Apparatus

The morphologies of the samples were recorded by transmission electron microscopy (TEM) and high-resolution transmission electron microscopy (HITACHI H-7650, Japan). Scanning electron microscope (SEM) images were obtained by a Scanning electron microscope (JSM-6300, Japan). XPS measurements were performed on a Thermo ESCALAB 250 using a monochromic Al X-ray source (1486.6 eV). All the electrochemicals data were measured by CHI 760D electrochemical workstation (Shanghai Chenhua, China). Fourier transform infrared (FTIR) spectra of the SiO_2_ NPs and SiO_2_-PA NPs were obtained from a VARIAN Cary 5000 Fourier transform infrared spectrophotometer (VARIAN, USA). Surface potential of the samples was performed by Zeta potential analyzer (Malvern Instruments ZS90). All experiments were carried out using a three-electrode cell equipped, which consisted of a platinum electrode, saturated calomel electrode (SCE) and working electrode.

### Culture of Cells

The HeLa cells were cultured in Dulbecco’s Modified Eagle’s medium (DMEM) containing 10% fetal bovine serum (FBS), 100 units·mL^−1^ penicillin, and 100 μg·mL^−1^ streptomycin at 37 °C. Then, the HeLa cells were centrifuged for the electrochemical experiments. Real sample measurements were performed by the addition of 100 μg·mL^−1^ PMA in PBS containing 50 mM glucose.

### Preparation of SiO_2_-Mn_3_(PO_4_)_2_ NPs

Firstly, SiO_2_ NPs were synthesized by the reverse microemulsion method as reported previously by Bagwe^48^. In a typical synthesis, triton X-100 (10.62 g), hexanol (9.6 mL) and cyclohexane (45 mL) were mixed in a 100 mL round-bottomed flask under stirring for 10 min, and then water (2.88 mL) was added to the mixture at room temperature. After being stirred for 0.5 h, NH_3_·H_2_O (600 μL) and TEOS (1200 μL) were dropped into the above clear solution, respectively. Next, the mixture was allowed to stir for a further 24 h at room temperature. The resulting NPs were collected by centrifugation and dried at 60 °C under vacuum condition for 24 h. Then, the modified process of SiO_2_ was briefly described as follows: SiO_2_ NPs (0.05 g) was dissolved in double-distilled water (20 mL), and TEOS (100 μL) was added to the SiO_2_ suspension with continuously stirring for 30 min at room temperature^49^. Then, the SiO_2_-NH_2_ NPs were obtained by feeding appropriate amount of APTES. After stirring for 30 min, 120 μL of PA/PA sodium salt hydrate buffer solution (pH = 7) was injected into the above solution with continues stirring for 24 h. The resulting NPs were washed with alcohol and double-distilled water. Finally, the SiO_2_-PA NPs were redispersed in water (10 mL), and then MnSO_4_ aqueous solution (10 mL, 12 mM) was injected to the round-bottomed flask containing the SiO_2_-PA NPs under constant stirring for 1 h at 60 °C. After completion of the reaction, the obtained products were collected by centrifugation, washed with double-distilled water, and dried in a vacuum oven at 60 °C for 24 h.

### Fabrication of SiO_2_-Mn_3_(PO_4_)_2_/MWCNTs/GCE

Firstly, MWCNTs (8.0 μL, 2.5 mg·mL^−1^) were cast onto the electrode surface and dried at room temperature. After that, SiO_2_-Mn_3_(PO_4_)_2_ (8.0 mg·mL^−1^) and 2.5% Nafion with the volumetric ratio of 1:1 were mixed, and 8.0 μL of above solution was dropped onto the surface of MWCNTs/GCE. After drying in air, the SiO_2_-Mn_3_(PO_4_)_2_/MWCNTs/GCE was obtained. During the experimental period, the modified electrodes were stored at 4 °C until use.

### Generation of superoxide anion

A stable O_2_^·−^ solution was prepared by dispersing KO_2_ to DMSO (containing 18-crown-6). In accordance with the molar absorptivity of O_2_^·−^ in DMSO, the concentration of O_2_^·−^ was monitored by recording the absorbance of ferricytochrome c spectrophotometrically at 550 nm^50^. In particular, spectrophotometric measurement of the amount of ferricytochrome c that reduced by O_2_^·−^ referred to the following reaction: cytochrome c (Fe^III^) +O_2_^·−^ = ferrocytochrome c (Fe^II^) +O_2_, in which ferrocytochrome c exhibits a strong absorbance at 550 nm^51^. The linear relationship of the absorbance *vs* the ferrocytochrome c concentration was depicted in Figure S13. The linear equations were A_550_ = 20.6c − 0.0044, R = 0.9986. The concentration of O_2_^·−^ can be calculated by the concentration of the formed ferrocytochrome c according to the above reaction formula^52^.

## Additional Information

**How to cite this article**: Shen, X. *et al*. Manganese Phosphate Self-assembled Nanoparticle Surface and Its application for Superoxide Anion Detection. *Sci. Rep.* **6**, 28989; doi: 10.1038/srep28989 (2016).

## Supplementary Material

Supplementary Information
