# Supplementary material for: Manganese Phosphate Self-assembled Nanoparticle Surface and Its application for Superoxide Anion Detection
Source: Sci Rep. 2016 Jun 30;6:28989. doi: 10.1038/srep28989 (PMC4928044; doi:10.1038/srep28989)
Supplement: Supplementary Information [file srep28989-s1.doc]

Supporting Information

**Manganese Phosphate Self-assembled Nanoparticle Surface and Its application for Superoxide Anion Detection**

Xiaohui Shen, Qi Wang, Yuhong Liu, Wenxiao Xue, Lie Ma, Shuaihui Feng, Mimi Wan*, Fenghe Wang, Chun Mao*

National and Local Joint Engineering Research Center of Biomedical Functional Materials, Jiangsu Key Laboratory of Biofunctional Materials, School of Chemistry and Materials Science, Nanjing Normal University, Nanjing, 210023, China, +86 25 85891651.

E-mail: (maochun@njnu.edu.cn (C. Mao), wanmimi@njnu.edu.cn (M. Wan)).


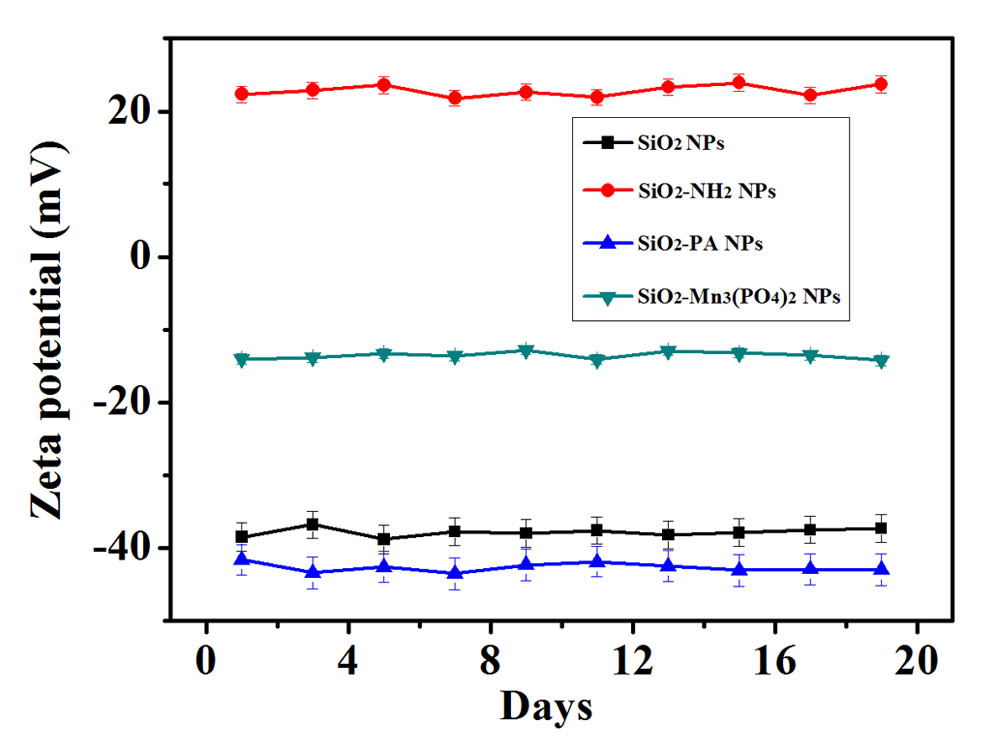


**Figure S1.** Zeta potential analysis.


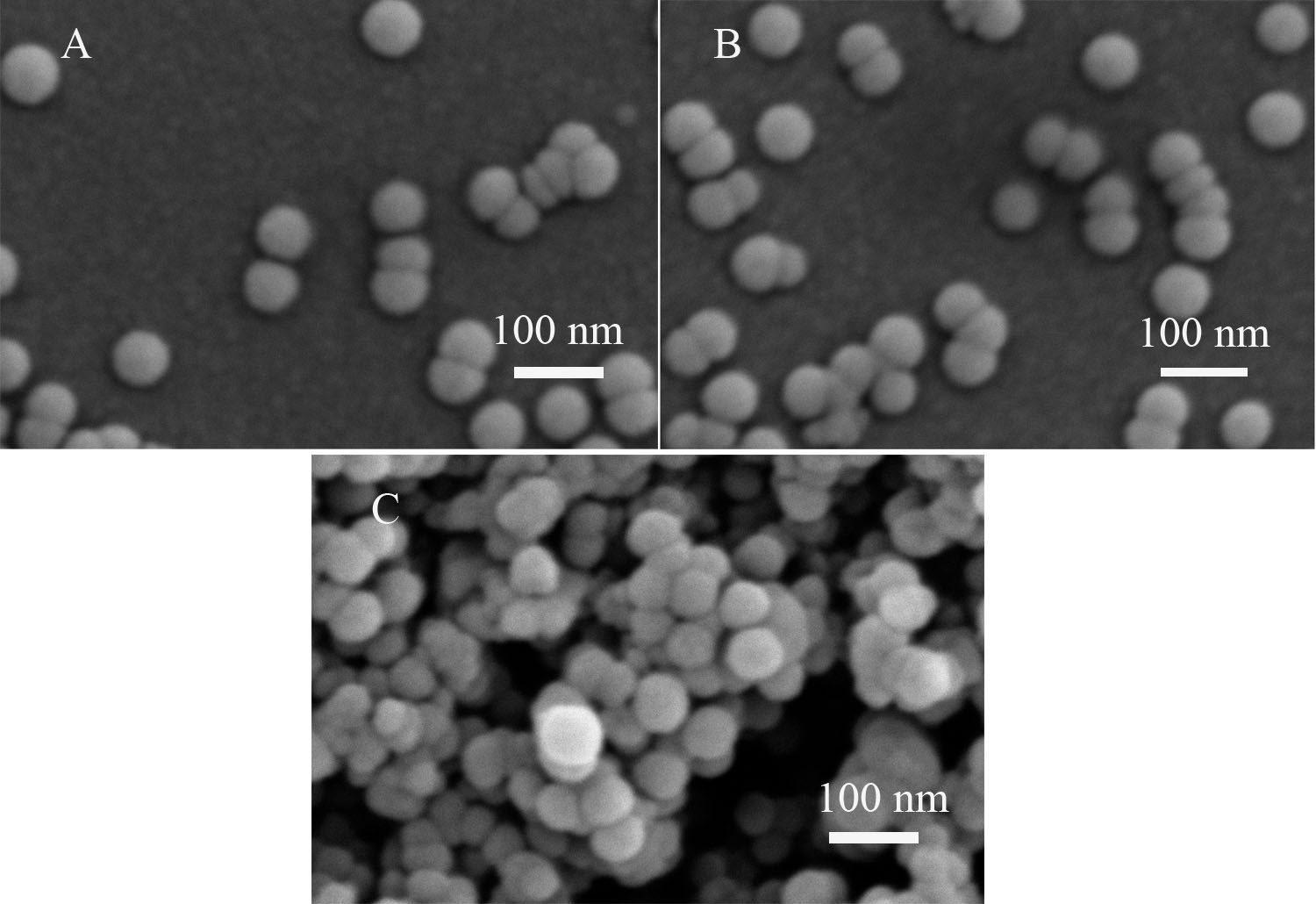


**Figure S2.** SEM images of (**A**) SiO_2_ NPs, (**B**) SiO_2_-PA NPs, and (**C**) SiO_2_-Mn_3_(PO_4_)_2_ NPs.


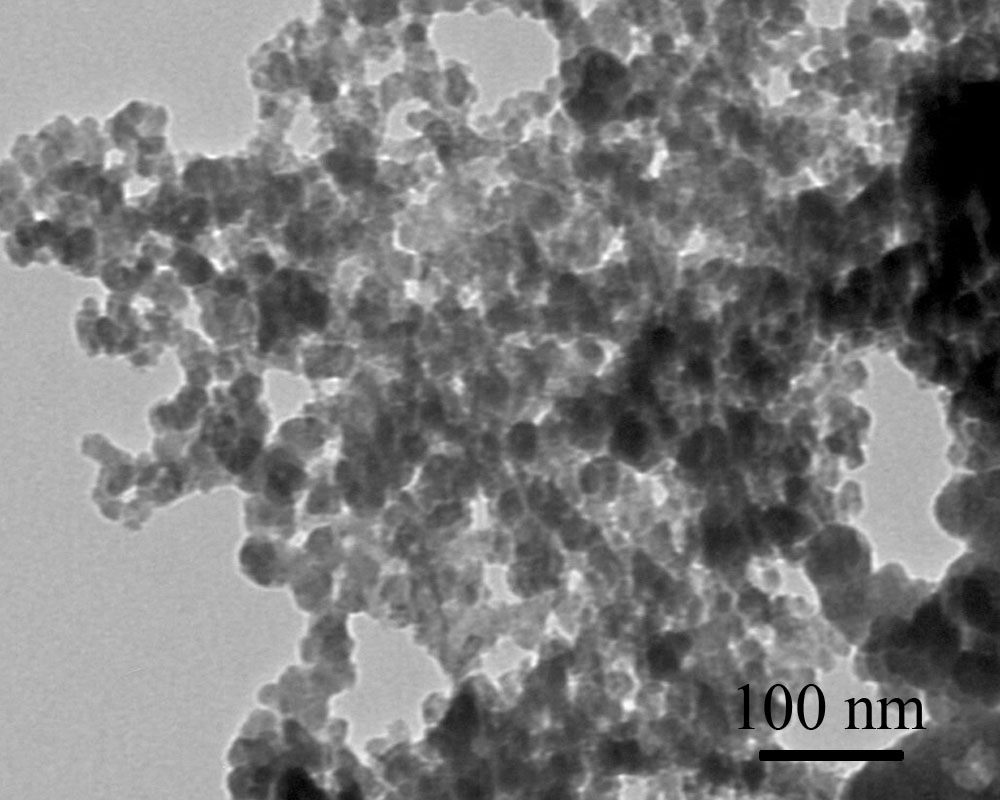


**Figure S3**. TEM images of nanoparticles Mn_3_(PO_4_)_2_ in the absence of SiO_2_ NPs with same heating and stirring conditions.


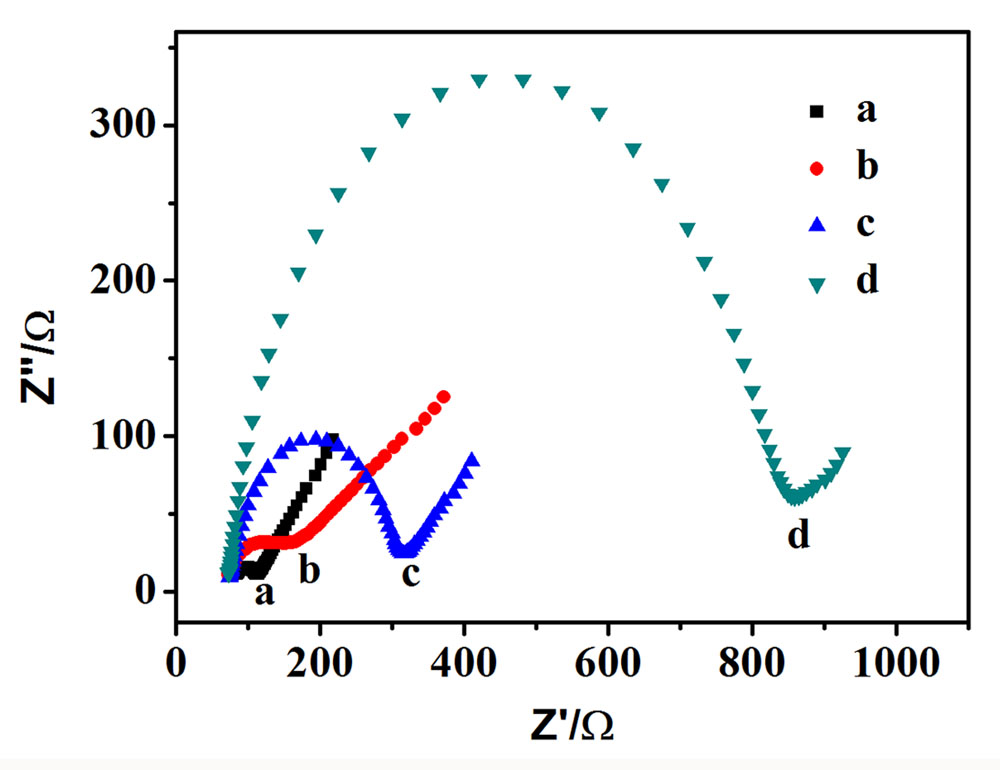


**Figure S4**. The Nyquist plots of (**a**) bare GCE, (**b**) MWCNTs/GCE, (**c**) SiO_2_-Mn_3_(PO_4_)_2_/MWCNTs/GCE, and (**d**) SiO_2_-Mn_3_(PO_4_)_2_/GCE in the presence of 10 mM of [Fe(CN)_6_] ^3–/4–^ and 0.1 M KCl.

Figure S4 showed the Nyquist diagrams of the bare GCE, MWCNTs/GCE, SiO_2_-Mn_3_(PO_4_)_2_/MWCNTs/GCE and SiO_2_-Mn_3_(PO_4_)_2_/GCE. The impedance spectrum of the bare GCE was showed in curve (a). When MWCNTs was covered on the GCE (curve b), there was a very small semicircle domain, indicating that MWCNTs exhibited excellent conductivity. After the SiO_2_-Mn_3_(PO_4_)_2_ was coated on the MWCNTs/GCE, the semicircle domain enlarged (curve c) due to the poor electrical conductivity of SiO_2_-Mn_3_(PO_4_)_2_ NPs. When only SiO_2_-Mn_3_(PO_4_)_2_ was modified on the GCE, the resistance of the composite dramatically increased (curve d).


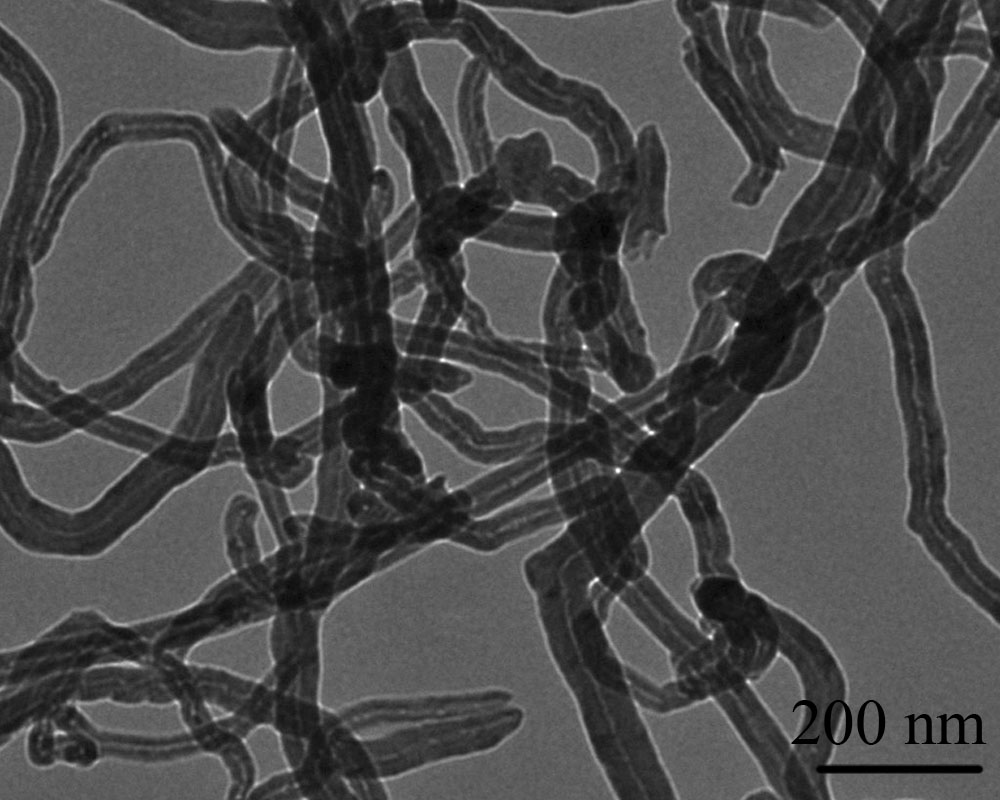


**Figure S5**. TEM images of Multi-walled carbon nanotubes.


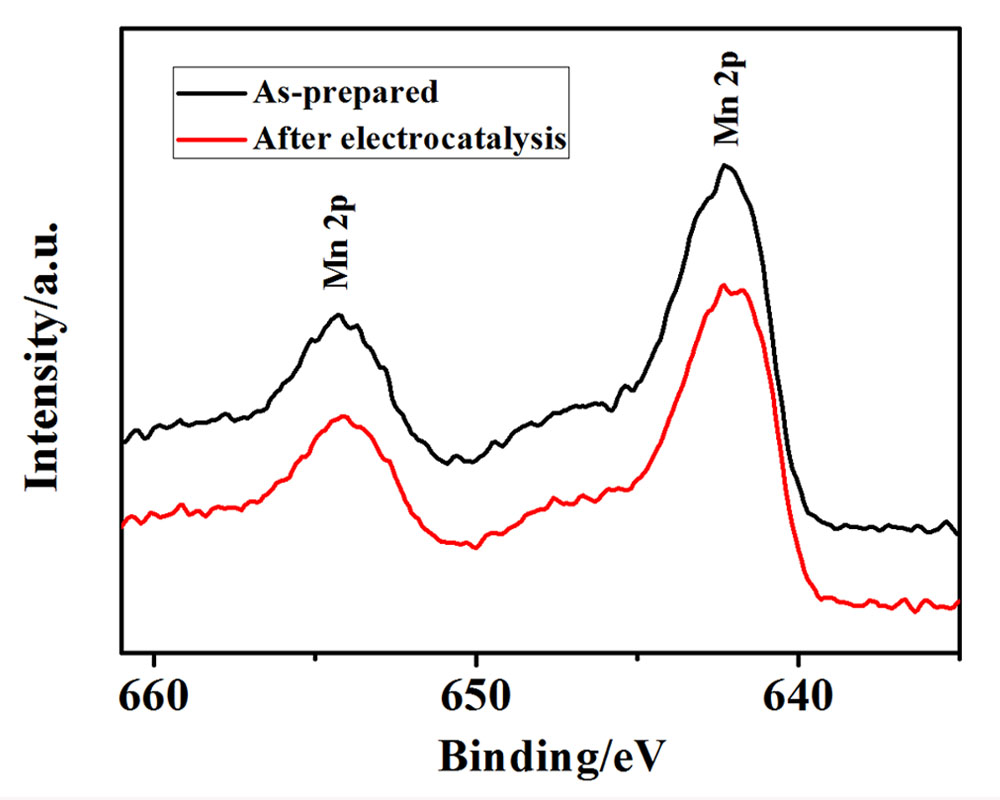


**Figure S6.** XPS spectra of the Mn 2p region of the as-prepared and after electrocatalysis SiO_2_-Mn_3_(PO_4_)_2_ at an applied potential of 0.484 V. The electrochemical reactions were performed in PBS solution (pH 7.4) upon successive additions of O_2_^•−^, scan rate: 100 mV·s^-1^.

XPS analysis was carried out to analyze the composition and chemical valence state of the SiO_2_-Mn_3_(PO_4_)_2_ NPs before and after electrocatalysis process. The XPS spectra of the Mn 2p of the as-prepared and SiO_2_-Mn_3_(PO_4_)_2_ after electrocatalysis was showed in Figure S6. As shown in Figure S6, Mn 2p spectra of the as-prepared sample contained two peaks centered at 642.3 eV and 654.5 eV that can attributed to Mn^2+^ reported by the previous paper^1^. After 1 h of electrocatalysis, the peak position of Mn 2p remained unchanged compared with that of the as-prepared sample, which was in accordance with many other literatures^2^. Based on the previous reports^3^, superoxide reacted rapidly with manganous phosphate complex to form a MnO_2_^+^ transient, which can hardly detect by XPS analysis for the final product. Direct studies by pulse radiolysis verified that superoxide reacted rapidly with manganous ion to form a short lived manganous-superoxide transient, MnO_2_^+^, which would change to Mn^2+^ in its subsequent reactions^2^.


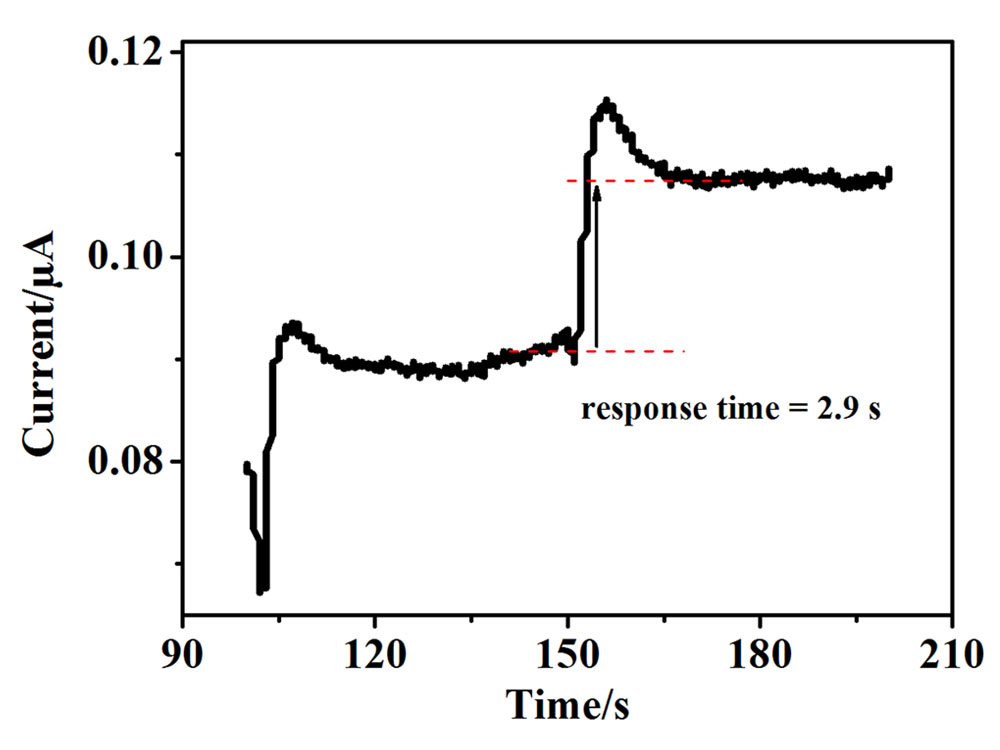


**Figure S7**. The response time record for successive injection of O_2_^•−^ at SiO_2_-Mn_3_(PO_4_)_2_/MWCNTs/GCE measured at 0.484V.


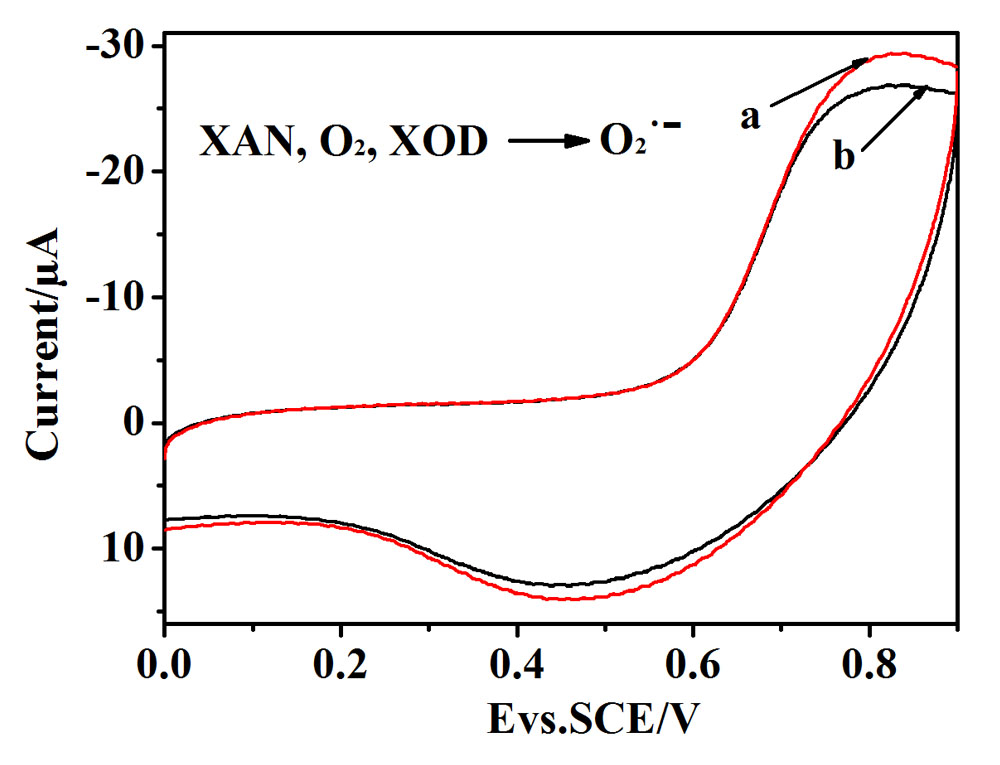


**Figure S8.** CVs of SiO_2_-Mn_3_(PO_4_)_2_/MWCNTs/GCE in the presence (**a**) and absence (**b**) of XAN/XOD in PBS solution (pH 7.4), scan rate: 100 mV·s^-1^.

The biosensor in the absence of XAN/XOD and presence of XAN/XOD in PBS solution were tested by CV, respectively. As shown in Figure S8, the biosensor in the absence of XAN/XOD and presence of XAN/XOD in PBS solution were tested by CV, respectively. Both anodic and cathodic peak currents of the PBS containing XAN/XOD (curve a) increased compared with that of PBS solution without XAN/XOD (curve b). Results indicated that the generated O_2_^•−^ could undergo automatic dismutation into O_2_ and H_2_O_2_ under the experimental conditions.


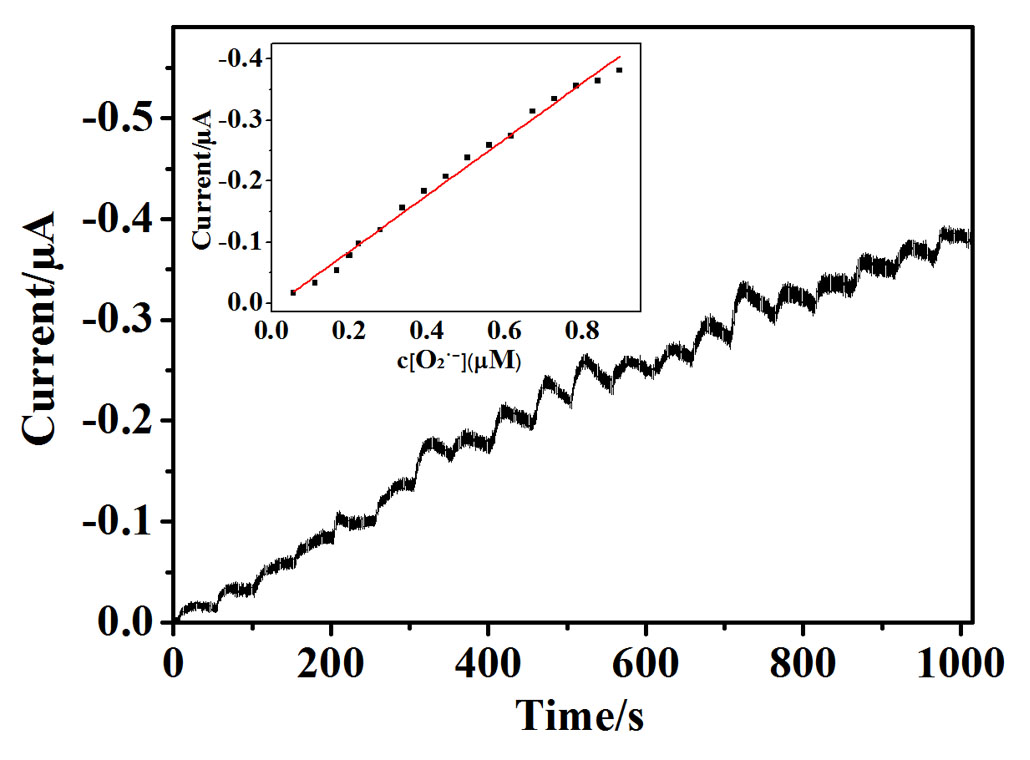


**Figure S9.** Amperometric response of SiO_2_-Mn_3_(PO_4_)_2_/MWCNTs/GCE upon successive XAN injection under stirring into 4 mL of 0.1 M pH 7.4 PBS containing 0.002 U mL^-1^ XOD. Inset: calibration curve for O_2_^•−^.

The response of SiO_2_-Mn_3_(PO_4_)_2_/MWCNTs/GCE toward O_2_^•−^ generated by XAN/XOD was investigated by amperometric measurements^4^. As shown in Figure S9, with successive additions of XAN to the solution, a stepwise increase of the current response was observed. A linear response range was obtained from 0.056 to 0.9 µM and the linear regression equation was I (μA)=0.00761-0.4605c (μM) (R=0.9914), where I was current and c was the concentration of O_2_^•−^, verifying the applicability of SiO_2_-Mn_3_(PO_4_)_2_ NPs in detection of O_2_^•−^ generated by other methods.


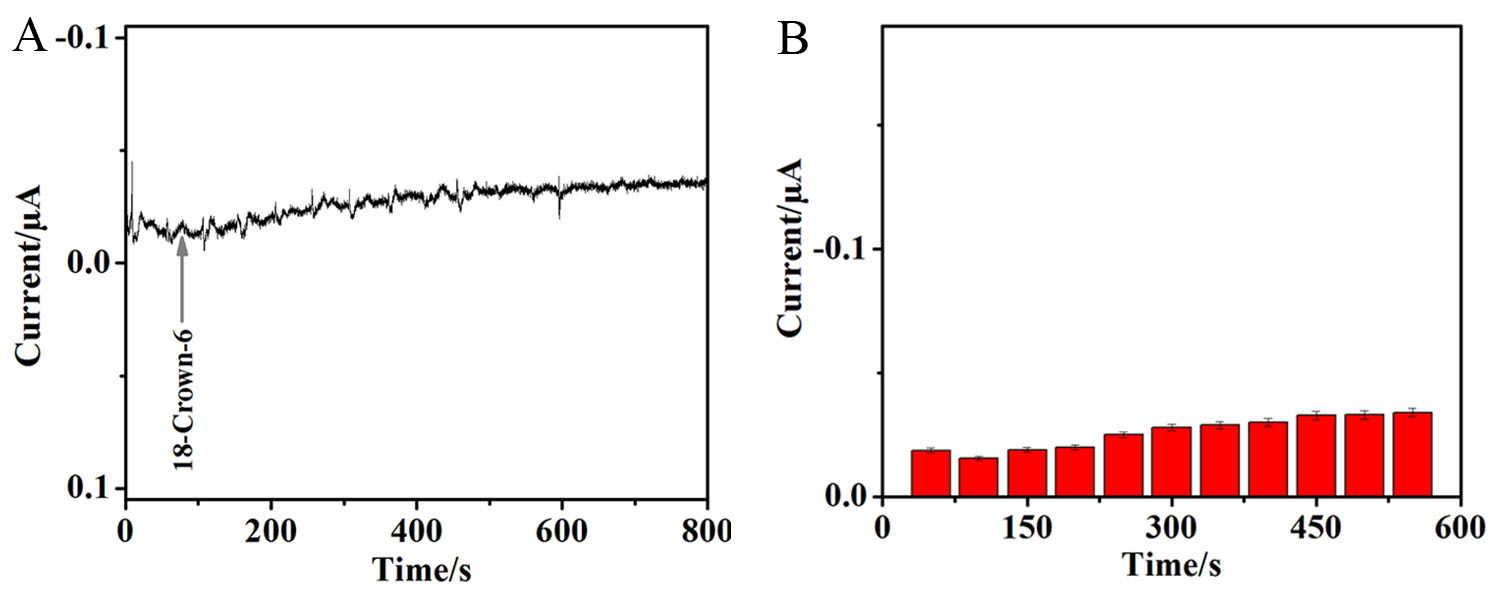


**Figure S10.** (**A**) Effect of 18-crown-6 on the detection system, and (**B**) The response current of the modified electrodes.


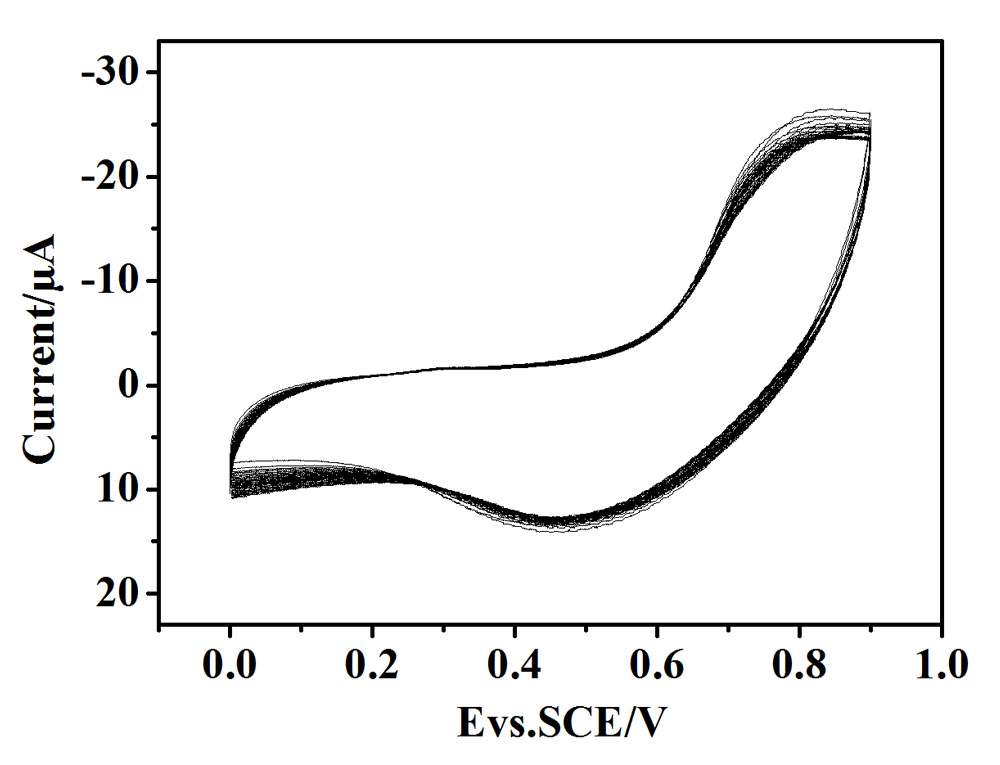


**Figure S11.** Cyclic Voltammetry (20 cycles) of SiO_2_-Mn_3_(PO_4_)_2_/MWCNTs/GCE in nitrogen saturated PBS at a scan rate of 100 mV·s^-1^.


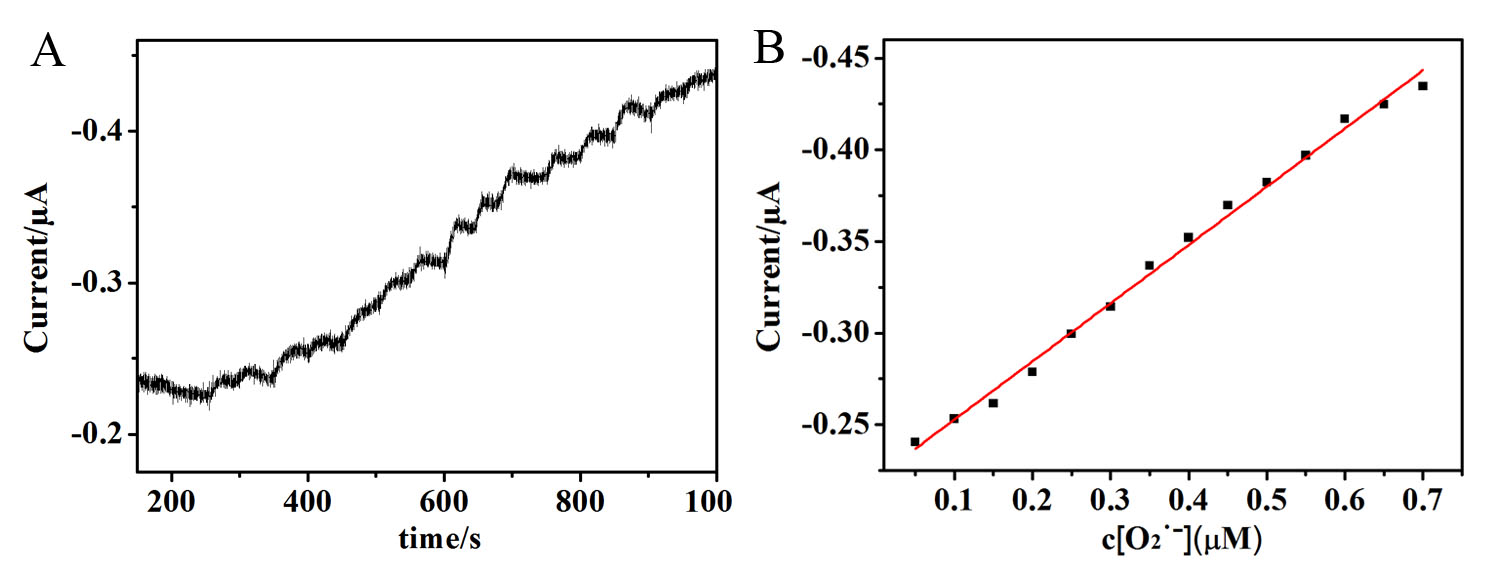


**Figure S12.** (**A**) Typical amperometric curve recorded at 0.484 V in plasma samples for SiO_2_-Mn_3_(PO_4_)_2_/MWCNTs/GCE, From 0.05 to 0.7 μM, the O_2_^•−^ concentration of each adding step was 0.05 μM, (**B**) Linear calibration plot of the response current vs the O_2_^•−^ concentration.

The O_2_^•−^ concentrations in plasma samples were determined using the standard addition method^5,6^. As shown in Figure S12, the amperometric responses of the SiO_2_-Mn_3_(PO_4_)_2_/MWCNTs modified GCE were investigated with the successive addition of O_2_^•−^ in 5 mL of plasma under intense stirring. Results indicated that with the increasing O_2_^•−^ concentration in a range from 0.05 to 0.7 μM, the peak current of catalytic oxidation was simultaneously increased linearly. The linear regression equation was I (μA)=-0.221-0.3178c (μM) with a correlation coefficient (R) of 0.9946. Results verified that this technology can be used to detect plasma.


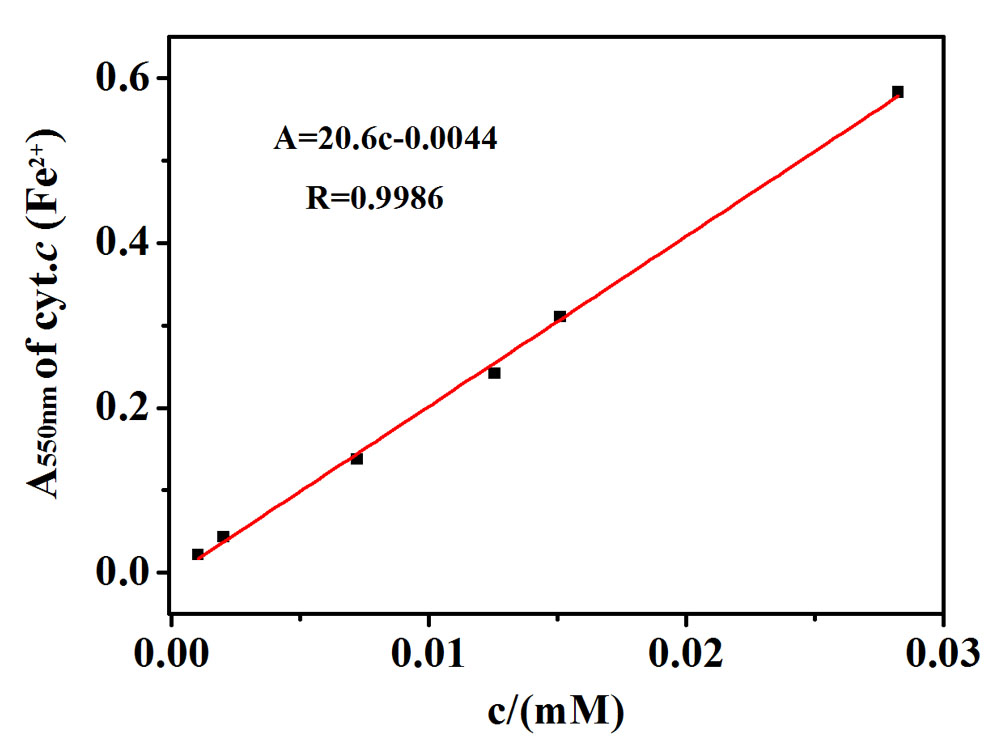


**Figure S13.** Linear standard curve of the absorbance vs the concentration of ferrocytochrome c.

**Table 1.** Comparison of detection limit of some O_2_^•−^ biosensors that reported by previous papers.

| Superoxide anion biosensor | Detection limit | Correlation coefficient | Ref. |
| --- | --- | --- | --- |
| KMWNTs-[BMIM]PF_6_-SOD/GCE | 0.024 μM | 0.998 | [7] |
| Mn^2+^/Nafion/TiO_2_ | 0.17 μM | 0.997 | [8] |
| SOD/Pt-Pd/MWCNTs/SPGE | 0.71 μM | 0.994 | [9] |
| Mn_2_P_2_O_7_-formylstyrylpyridine/GCE | 0.029 μM | 0.991 | [10] |
| Mn^2+^-ZSM/PDDA/GCE | 0.37 μM | 0.997 | [11] |
| SiO_2_-Mn_3_(PO_4_)_2_/MWCNTs/GCE | 0.0175 μM | 0.997 | This work |

**References**

1. Hu, F. X. *et al.* Living cells directly growing on a DNA/Mn_3_(PO_4_)_2_-immobilized and vertically aligned CNT array as a free-standing hybrid film for highly sensitive in situ detection of released superoxide anions. *Adv. Funct. Mater.* **25**, 5924-5932 (2015).
2. Barnese, K., Gralla, E. B., Cabelli, D. E. & Valentine, J. S. Manganous phosphate acts as a superoxide dismutase. *J. Am. Chem. Soc.* **130**, 4604-4606 (2008).
3. Luo, Y. P., Tian, Y. & Rui, Q. Electrochemical assay of superoxide based on biomimetic enzyme at highly conductive TiO_2_ nanoneedles: from principle to applications in living cells. *Chem. Commun.* **21**, 3014-3016 (2009).
4. Wang, L. *et al.* A novel amperometric biosensor for superoxide anion based on superoxide dismutase immobilized on gold nanoparticle-chitosan-ionic liquid biocomposite film. *Anal. Chim. Acta* **758**, 66-71 (2013).

# Sun C. *et al.* **Hemocompatible and antibiofouling PU-F127 nanospheres platform for application to glucose detection in whole blood.** *J. Mater. Chem. B* 1, 801-809 (2013).

# Sun C. *et al.* An innovative glucose biosensor using antibiofouling Au-F127 nanospheres. *J. Biomed. Nanotechnol*. 9, 1-8 (2012).

7. Li., Wang, B., Xu, J. J. & Chen, H. Y. In vitro detection of superoxide anions released from cancer cells based on potassium-doped carbon nanotubes-ionic liquid composite gels. *Nanoscale* **3**, 5026-5033 (2011).

8. Luo, Y. P., Tian, Y. & Rui, Q. Electrochemical assay of superoxide based on biomimetic enzyme at highly conductive TiO_2_ nanoneedles: from principle to applications in living cells. *Chem. Commun.* **21**, 3014-3016 (2009).

9. Zhu, X., Niu, X. H., Zhao, H. L., Tang, J. & Lan, M. B. Immobilization of superoxide dismutase on Pt-Pd/MWCNTs hybrid modified electrode surface for superoxide anion detection. *Biosens. Bioelectron.* **67**, 79-85 (2015).

10. Yuan, L. *et al.* Biomimetic superoxide dismutase stabilized by photopolymerization for superoxide anions biosensing and cell monitoring. *Anal. Chem.* **86**, 4783-4790 (2014).

11. Zhou, J. Q. *et al.* A reliable and durable approach for real-time determination of cellular superoxide anion based on biomimetic superoxide dismutase stabilized by a zeolite. ***Analyst*** **136**, 1594-1598 (2011).
